# Special Issue: Honey Bee Pathogens and Parasites

**DOI:** 10.3390/vetsci9100515

**Published:** 2022-09-21

**Authors:** Julia Ebeling, Anne Fünfhaus, Sebastian Gisder

**Affiliations:** Department of Molecular Microbiology and Bee Diseases, Institute for Bee Research, 16540 Hohen Neuendorf, Germany

Honey bees are important pollinators of agricultural crops and despite the reports about elevated local colony losses over the last few decades [1,2,3,4], the number of honey bee colonies has increased on a global scale [5,6]. However, the demand for pollination service has increased in a way that is disproportional to the increase in honey bee colonies, which will probably lead to a global pollination crisis [7,8]. In this regard, colony losses caused by honey bee pathogens and parasites are particularly alarming. The first description of diseases of the honey bee can be found in “*The history of animals*” by Aristotle (384–322 BC), which refers to an infestation with wax moths and the first description of foulbrood (“The diseases that chiefly attack prosperous hives are first of all the clerus-this consists in a growth of little worms on the floor, from which, as they develop, a kind of cobweb grows over the entire hive, and the combs decay; another diseased condition is indicated in a lassitude on the part of the bees and in malodorousness of the hive.”) [9]. Until now, honey bee health has been threatened by a multitude of pathogens and parasites. Important examples of the threats to managed honey bee colonies are infestations with mites, such as *Varroa destructor* and *Tropilealaps* spp., or the small hive beetle *Aethina tumida*, infections with fungi (*Ascosphaera apis*) and microsporidia (*Nosema apis* or *Nosema ceranae*), protozoans (*Malpighamoebae mellificae*), bacteria (*Paenibacillus larvae* or *Melissococcus plutonius*), and viruses (e.g., deformed wing virus and acute bee paralysis virus). This is why constant and state-of-the-art research on detection methods, pathology, epidemiology, and development of treatment strategies is indispensable in the field of honey bee pathogens and parasites. In this Special Issue “Honey Bee Pathogens and Parasites”, we present original research articles, research communication on new detection methods, and review articles on the current state of the art for molecular pathogen detection and alternative treatment methods.

The examination of honey for environmental DNA (eDNA), i.e., traces from organisms left in the honey, is a useful tool to identify pathogens that are not directly visible, but have previously been in contact with the honey matrix. Ribani and colleagues developed a PCR method that targets the mitochondrial gene cytochrome oxidase I for the specific detection of environmental DNA of the greater wax moth *Galleria mellonella* and the small hive beetle *Aethina tumida* in honey. This method will be useful for the early identification and monitoring of the spread of these two honey bee pests [10].

The detection of the pathogen *Malpighamoeba mellificae,* which causes amoebiasis disease in honey bees, was enabled via a RT-qPCR method based on 18S rRNA sequences. The protozoan infects the Malpighian tubules responsible for excretion and osmoregulation. The new detection method enables identification and monitoring for an improvement in honey bee health [11].

Sacbrood virus (SBV) causes disease in honey bee larvae from the European honey bee *Apis mellifera*, the Asian honey bee *Apis cerana* (Ac), and the Asian hornet *Vespa velutina*, being most severe in *A. cerana*. The detection of all present SBV variants cannot be ensured with the existing quantitative PCR methods. In a study by Blanchard et al., 2014, the TaqMan qPCR method was improved with new primers and an MGB (minor groove binder) probe. The method now enables the detection of all currently published SBV and Ac SBV genomes [12]. 

The early diagnosis of honey bee diseases is important to avoid further spread of diseases and economic losses to the beekeeper. The review article by Lannutti et al. gives an overview of the molecular methods for the detection and differentiation of arthropod, fungal, protozoan, bacterial, and viral honey bee pathogens [13].

Marín-García and colleagues discuss the role of the microsporidium *Nosema ceranae*, the etiological agent of type C nosemosis, in honey bee colony losses. Furthermore, the article reports the efficacy of plant extracts, nutraceuticals, probiotics, propolis, and veterinary drugs as alternative treatment strategies. They might be a substitute for the commonly used antibiotic fumagillin, which is prohibited in the European Union [14]. 

Entomopathogenic fungi may represent a further possibility for pest and predator control, as presented by Bava et al. Entomopathogenic fungi are being investigated as biocontrol agents against *V. destructor*, *A. tumida*, and *Vespidae*. The search for alternatives in pest control is especially important, due to the emergence of resistance to the existing chemical drugs [15]. 
